# An Update on Medical School Accreditation in the United States: Implications for the Single Graduate Medical Education (GME) Era

**DOI:** 10.7759/cureus.34884

**Published:** 2023-02-12

**Authors:** Harris Ahmed, Michael Kortz, J. Bryan Carmody

**Affiliations:** 1 Ophthalmology, Loma Linda University Medical Center, Loma Linda, USA; 2 Neurosurgery, University of Colorado, Denver, Colorado, USA; 3 Pediatric Nephrology, Eastern Virginia Medical School, Norfolk, USA; 4 Pediatric Nephrology, Children's Hospital of The King's Daughters, Norfolk, USA

**Keywords:** single accreditation, lcme-accredited, lcme, coca, osteopathic medical schools, medical school education

## Abstract

In the United States, medical schools are accredited by either the Liaison on Committee Medical Education (LCME) or the Commission on Osteopathic College Accreditation (COCA), which assesses the quality and standards of Doctor of Medicine (MD)-granting and Doctor of Osteopathic Medicine (DO)-granting institutions, respectively. Thereafter, new MD and DO physicians complete graduate medical education (GME) training. Historically, the two physician licensure pathways have been predominantly separate, but in 2020, the Accreditation Council for Graduate Medical Education and American Osteopathic Association finalized a single accreditation GME system. Now, other elements of MD and DO physician training that have traditionally remained separate, such as undergraduate medical education (UME), are increasingly being scrutinized. Since 2010, when the accreditation of UME was last qualitatively criticized, the standards and competencies set forth by LCME and COCA have converged. COCA, in particular, has updated its requirements to emphasize scholarly activity, improve inpatient clinical rotation requirements, engage medical students, and enhance clinical faculty qualifications. Such convergence brings to question the continuing need for two independent accreditation pathways and barriers that may prevent a single accreditation. We argue that although MD and DO physicians are unique, the natural confluence of UME accreditation represents an opportunity to simplify and improve physician training in the United States. Our analysis suggests the major barriers to implementing a single accreditation system surround the requirement of Osteopathic Manipulative Medicine (OMM)-focused faculty by COCA and the two separate licensing exams (USMLE (United States Medical Licensing Examination) and COMLEX (Comprehensive Osteopathic Medical Licensing Examination)). However, with a continuing decline in osteopathic physicians practicing OMM and growing debate over a new single licensing exam, a single accreditation UME system may be practically achieved.

## Editorial

The process of national accreditation for medical schools in the United States is believed to have begun in 1847 after a meeting in Philadelphia with the American Medical Association (AMA). During this meeting, it was determined that a national convention would elevate the standards of medical education in the United States [1]. The parties initially involved also included allies of the AMA such as the American Academy of Medicine and the Association of American Medical Colleges (AAMC). A few years after this meeting, modern undergraduate medical education in the United States as we know it today gained footing in 1910 after Abraham Flexner released his scathing report on schooling quality across the nation [2]. A few decades later, the Liaison Committee on Medical Education (LCME) was founded at a 1942 meeting between leaders of the AAMC and the AMA [3]. One of the driving forces behind the establishment of this new entity to accredit medical schools was to minimize duplication between the AAMC and AMA in accrediting medical schools and provide a more unified front for accreditation in the United States [3].

Although the library of clinical knowledge has exponentially grown since these early landmark moments, students still typically progress through medical school curricula in four years. There are two degrees that grant licensed physicianship in the US: Doctor of Medicine (MD) and Doctor of Osteopathic Medicine (DO) [4]. MD and DO graduates have become increasingly similar in clinical training and practice, especially as the proportion of DOs who practice osteopathic manipulative treatment (OMT) has decreased [5]. With the single accreditation system for graduate medical education (GME) finalized as of 2020, the attention has turned to other different aspects of MD and DO medical education [6].

One area where MD and DO education remains separate is in medical school accreditation. The accreditation process is in place to ensure the public and prospective students that institutions meet a minimum level of quality. The American Association of Medical Colleges’ Liaison on Committee Medical Education (LCME) accredits MD-granting schools while the American Osteopathic Association’s Commission on Osteopathic College Accreditation (COCA) accredits DO-granting schools [7,8]. With the changing needs of a dynamic population, defining and validating the quality of medical education remains as important as ever [9-11].

In 2009, Wood and Hahn evaluated LCME and COCA standards and organizational structures, finding substantial similarities [12]. However, there were important differences noted in the ways members were chosen, the ability to start investor-owned medical schools, and standards related to academic/research output [12]. This comparison generated significant discussion and dialogue, however, the medical school accreditation process has not been critically evaluated since [12,13]. Herein, we aim to provide an update on the accreditation processes and standards of the LCME and COCA and discuss the possibility of a single medical school accreditation system.

Accreditation standards: comparing LCME and COCA standards

Governance and Administration

When Wood and Hahn last compared COCA and LCME standards in 2009, the latter asserted that for-profit schools would only be accredited under extraordinary and justifiable standards [12]. However, both the LCME and COCA now accredit these investor-owned medical schools. In general, for-profit MD and DO schools’ tuition is comparable to a majority of non-profit institutions, with similar graduation, attrition, and residency placement rates [14,15].

The previous review documented functional similarity in governance for both bodies, though with one important exception in the lack of student representation in COCA [12,13]. However, COCA now has student representation on various committees, including a board position with full voting rights; otherwise, the basic structure of both accrediting bodies remains functionally similar.

In the brief period of their existence, for-profit medical schools (either MD or DO) appear not to jeopardize the quality or affordability of physician training in the United States in comparison to non-profit institutions, but the long-term outlook remains to be seen. There continues to be debate surrounding this topic, especially in the context of loans, tuition, and interest rates.

Because the LCME and COCA standards are so similar, little would have to be reconciled to create a single accreditation system. A single accrediting body would need to ensure fair and adequate representation of MD- and DO-granting schools in its governance, however. 

Academic and Learning Environment

Differences between the LCME and COCA academic environment standards have become conspicuous. In the LCME standards, there are six provisions under the “Academic and Learning Environments” section. These include guidelines for resident participation in medical student education, scholarship and research opportunities, diversity promotion, anti-discrimination, professionalism, and mistreatment of medical students. COCA standards outline four provisions under the “Learning Environment” section, specifying requirements for professionalism, diversity promotion, safety and wellness, and patient care supervision.

Previously, LCME and COCA standards differed significantly in regard to the LCME’s requirement that clinical education occurs at training sites with GME. However, COCA standards also now mirror that of LCME regarding resident participation in medical student education, where students must be taught in at least one clinical environment in the presence of a GME program, as well as receive relevant inpatient experience [7-8]. Not requiring the presence of residents at every training site allows for medical schools to exist in rural areas without large medical centers, this has been critical for many DO schools but also MD schools such as Florida State.

Both LCME and COCA standards leave substantial room for interpretation in how academic and learning environments, especially in the clinical setting, should be provided for students. Most standards are broadly similar. The logistics for these standards, especially for DO-granting institutions, are of particular interest given that most do not have major academic hospital affiliates and rather rely on community engagement, private preceptorships, and contractual agreements with health systems. Neither document specifies whether or how nonphysician providers, such as nurse practitioners, physician assistants, and certified registered nurse anesthetists, should be utilized in medical student training.

Exploring the role of nonphysician providers and practical implementation of the above academic and clinical standards represent a great opportunity for LCME and COCA leadership to join together to guide the future of medical education in these regards.

Faculty and Staff

Another major change, per Element 6.9c, COCA now requires that individual schools must provide adequate clinical faculty (defined as a three-year rolling average of the number of eligible second-year and repeating students) at each COM [16]. Both LCME and COCA require clinical faculty to be board certified and require sufficient resources and support staff to promote the mission of the institution and well being of its students. Both documents promote faculty development programs, however, only LCME encourages that faculty “demonstrate a commitment to continuing scholarly productivity that is characteristic of an institution of higher learning” [7]. Only COCA requires there to be a primary care physician in leadership and specific osteopathic manipulative medicine (OMM) clinical faculty and notes that clinical departments can be structured as the medical school sees fit provided they meet other requirements [8].

There are no major differences between the COCA and LCME standards to make determinations of quality. However, given that most DO-granting and some MD-granting institutions do not have academic medical center affiliates and large research budgets, the ability for students to engage with specialty faculty and research under the guidance of a scholarly-driven mentor early during medical school may be limited compared to their peers. Neither document provides guidelines for how medical schools should staff surgical and subspecialty faculty beyond what is required for clinical education.

In terms of single accreditation, faculty and staff represent a major area that will require significant discussion. There remains a delicate balance between spending dense resources building expansive and diverse academic departments vs limiting the fiscal strain of schools located in under-resourced settings. Navigating this balance will require innovative solutions. The coronavirus 2019 (COVID-19) pandemic gave rise to many virtual clinical, research, and educational platforms, and perhaps there is an opportunity to mitigate gaps in faculty and staff for schools in under-resourced settings utilizing tech. Additionally, the inclusion and requirements for faculty versed in OMM would be another area requiring dialogue, with a reasonable solution allowing individual schools to determine the extent of their involvement in the practice of OMM.

Students

Both LCME and COCA require delineated admission policies as well as provide vague guidelines on which students to admit and how to accept transfer students. LCME requires there to be an appointed admission committee while COCA does not. LCME also provides guidelines on how to approach visiting students while COCA does not [7,8].

The standards between LCME and COCA regarding student policies and admission are similar. The lack of COCA guidelines on visiting students likely reflects the lack of in-house GME at DO-granting institutions. Given that DO schools tend to emphasize primary care and clinical training over research, subspecialty education, and scholarly activity, the COCA standards may benefit their institutions in providing guidelines on student selection with regard to these areas. Otherwise, these minor differences do not likely translate to quality discrepancies among student enrollment and could be overcome in a single accreditation process.

Educational Programs and Curriculum

COCA requires students to pass the first two levels of a licensing exam (COMLEX) while LCME does not require students to pass any level of a licensing exam (i.e., USMLE (United States Medical Licensing Examination) or COMLEX (Comprehensive Osteopathic Medical Licensing Examination)) as a requirement prior to graduation. While just over 85% of MD schools individually mandate the passage of USMLE Step 1 for promotion, it is not an LCME requirement [17]. Both sets of standards require the development of educational objectives which should guide the delivery of the curriculum [7,8].

While in theory LCME allows students to graduate without a licensing exam, practically MD schools have established licensing exams as a requirement. Before USMLE Step 1 became pass/fail, there was a growing trend among DO schools to mandate both USMLE in addition to COMLEX. There has now been growing discussion regarding a new unified licensing exam for LCME and COCA-accredited schools, making this an area to watch closely over the next few years [18-20].

If the two accreditation bodies were to merge, it is unclear if COMLEX would remain a requirement for historically COCA-accredited institutions, or if the requirement of passing licensing examinations would be removed, leaving the matter to individual schools. In any case, this represents an area where a decision and discussion would be required.

Research

A major change in recent years is that COCA now dedicates language to the promotion of research and scholarly activity. This includes provisions for promoting strategic plans and procedures for research and teaching the scientific method, outlining budgetary policies, and supporting students in their research endeavors. COCA standards also require medical schools to engage in OMM research as part of their budgetary and logistic priorities. LCME has long had research and scholarly activity a significant part of their guidelines, with special regard to budgetary priorities, requirements of faculty and staff, promotion of students’ scholarly output, and emphasis on a general environment of research. LCME does not require OMM research of its constituents [7,8].

Although COCA now promotes research and scholarly activity in their standards, there is little in the way of guidelines for how this should be implemented and to what degree scholarly activity is emphasized in budgets and resource allocation. LCME in general accredits institutions with larger endowments and greater grant and philanthropic research funding than COCA, which could certainly translate to less output from DO students. While DO schools tend to emphasize clinical training rather than research, this likely remains a significant hurdle for some osteopathic medical students who wish to pursue research-related careers.

In the scenario of single accreditation, it is likely that historically COCA-accredited schools would continue to produce less research output compared to historically LCME-accredited schools. At the graduate medical education level, the issue of research and scholarly activity was an issue for a subset of former AOA-accredited residency programs leading to the closures of surgical subspecialty programs in high-need areas. Lessons can be learned from that experience so as to avoid the closure of institutions in high-need areas due to scholarly activity requirements [21].

Approach to Diversity

Importantly, COCA has transitioned some of its diversity promotion languages from passive anti-discriminatory statutes to more proactive inclusionary statements in line with LCME standards [7,8].

This is an area where the house of medicine needs to progress across the board, with active revisitation and enhancement of standards, as research demonstrates that a more diverse physician workforce translates to better outcomes for underrepresented communities [22].

Convergence of MD and DO Education: A Single System?

Since 2010 when the accreditation of UME was last qualitatively criticized, the standards and competencies set forth by LCME and COCA have converged. COCA, in particular, has updated its requirements to emphasize scholarly activity, improve inpatient clinical rotation requirements, engage medical students, and enhance clinical faculty qualifications. The line between MD and DO education appears to be diminishing at the undergraduate medical education level as has occurred with GME. When analyzing the accreditation environment, COCA appears to be becoming more like the LCME, even in comparison to 10 years ago. Therefore, the question arises if there is still a need for two separate accreditation organizations and systems. Some may argue that maintaining COCA as an accrediting body remains important as historically, DO schools tend to be located in high-need areas, recruit students from high-need areas, place students in high-need areas for residency, and DO grads tend to serve underserved areas, all to a greater extent than MDs [23-27]. The physician shortage is of significant concern, especially in resource-limited areas [28]. Given that there are fewer barriers to the creation and accreditation of DO schools, such institutions have been easier to open in high-need environments.

If there is to be a single accreditation system between LCME and COCA, the ability of COCA standards to better facilitate the creation of medical schools in high-need settings through less directive research and academic department requirements, and more contracted and preceptor-based clinical rotations should be accommodated as best as possible as was done during the single accreditation system which was finalized in 2020 at the GME level. During the single accreditation system process at the GME level, there was fear among some stakeholders that the majority of osteopathic GME programs would fail to meet transition standards, however, a vast majority of former AOA programs successfully transitioned to the new ACGME single accreditation system [29]. Combining both organizations’ strengths and promoting an equitable and consistent environment across all schools will standardize and improve the quality of United States medical education across the board. If there were a single accreditation system, certain standards would have to be reconciled to continue accreditation for schools that have achieved accreditation under either system absent a material change in the school that would result in a change to accreditation status. Our analysis suggests major areas would include the issue of licensing exams (USMLE and COMLEX) and OMM staff and faculty requirements. With the growing debate on single licensing exams and the exponential decrease in osteopathic physicians utilizing OMM, it does not appear these areas represent major barriers to implementing single accreditation at the undergraduate medical education level. Using the GME single accreditation system as an example, there is a strong precedent for the successful unification of allopathic and osteopathic training pathways.

The natural confluence of UME accreditation represents an opportunity to simplify and improve physician training in the United States. Our analysis suggests the major barriers to implementing a single accreditation system surround the requirement of Osteopathic Manipulative Medicine (OMM) focused faculty by COCA and the two separate licensing exams (USMLE and COMLEX). However, with a continuing decline in osteopathic physicians practicing OMM and growing debate over a new single licensing exam, a single accreditation UME system may be practically achieved.
